# Predictors associated with HIV/AIDS patients dropout from antiretroviral therapy at Mettu Karl Hospital, southwest Ethiopia

**DOI:** 10.1186/s13104-019-4267-3

**Published:** 2019-04-18

**Authors:** Melaku Tadege

**Affiliations:** Department of Statistics, Injibara University, Injibara, Amhara Ethiopia

**Keywords:** AIDS, ART, Ethiopia

## Abstract

**Objective:**

The aim of this study was to determine the major risk factors of antiretroviral therapy dropout. The retrospective cohort research design was applied. 1512 HIV patients were included from Mettu Karl Hospital in Illubabor Zone, southwest part of Ethiopia from September 2005 to January 2018. Kaplan–Meier comparison and log-logistic regression accelerated failure time model were used.

**Results:**

From the log-logistic regression result, the risk of dropout for patients with primary education status was 10.58% greater as compared to illiterate (p < 0.0110). The probability of dropout for patients with marital status separated was about 16.82% higher than those patients with marital status divorced (p < 0.0070). Being merchant, farmer and daily labour had a greater risk of dropout as compared to a housewife. Most of the HIV/AIDS patients on ART were dropout in a short period due to patients separated marital status, primary education, CD4, being merchants, farmer and daily labour. Investigation on the cause of antiretroviral therapy dropout from a number of AIDS clinics in the country is highly appreciated.

**Electronic supplementary material:**

The online version of this article (10.1186/s13104-019-4267-3) contains supplementary material, which is available to authorized users.

## Introduction

HIV is the most responsible causes of mortality worldwide and the primary predictor of death in sub-Saharan Africa region. The prevalence of new infections in the area accounted for 66.6% of the world. Above 68% of adults and 90% of children infected with the disease were found in this area, and more than 76% of HIV/AIDS-related deaths were occurred in Africa [1]. In sub-Saharan Africa more than 2.2 million people were died per year due to HIV/AIDS and related causes [2, 3].

In Ethiopia, 780, 000 HIV/AIDS patients were on antiretroviral therapy [4] and around one million people are reportedly living with HIV. Of all people who have ever been reported as beginning antiretroviral treatment, 249,174 are adhering to their treatment regimen and there were 55,200 AIDS-related deaths in 2013 [5].

Antiretroviral therapy dropout is a serious challenge to the success of HIV/AIDS treatment. According to the world health organization report, from all patients enrolled in HIV, the percentage of success was only 23% [6]. Antiretroviral therapy dropout negatively affects the improvement of an immunological advantage of antiretroviral therapy and increases HIV/AIDS-related mortality [7]. Dropout of patients receiving antiretroviral therapy will be the reason for drug toxicity, treatment failure due to poor adherence, and drug resistance [8–10] this directly leads to death [11–15]. 40% of all patients on antiretroviral therapy were dropout in sub-Saharan Africa [16, 17]. Of all dropout patients in the region of sub-Saharan Africa, 46% of them were died [16].

Antiretroviral therapy can reduce HIV replication and it develops the immune ability [18]. There are limited data accesses about the results of the ART in Ethiopia. In Oromia region, there were 194,370 HIV/AIDS patients and of the 115,334 were on antiretroviral therapy. Of them, only 59.3% of HIV/AIDS patients were on ART which was far from adequate [19]. Another investigation also explained that the rate of antiretroviral therapy failure in private health facilities in Ethiopia was 20.4% [20]. In Jimma, one out of five adults had to antiretroviral therapy dropout which is a disaster for once country which aims to minimize the effect of HIV/AIDS [21].

HIV/AIDS patients with poor antiretroviral therapy follow up outcome are at high risk of death by two times than patients with good follow up adherence [22]. Patients who have poor follow up status were at risk of death by four times than who have well-adhered patients in Addis Ababa [23]. The risk of death of poor adhered patients is five times greater than better-adhered patients [24]. The study in Ethiopia also showed that around 50% of the antiretroviral therapy dropout patients were dead [25]. HIV/AIDS Patients who dropout antiretroviral therapy will likely die in a short period of time [26]. Ethiopia is among one of the most HIV/AIDS prevalence countries globally. ART treatment has a great role to prolong the life of HIV patients but, there were a high percentage of dropouts from antiretroviral therapy which causes directly facilitate death [27–29]. A study which was conducted in the Illubabor Zone recommended that investigation on antiretroviral therapy dropout in the area is timely [30]. Therefore, the aim of this study was to determine predictors of antiretroviral therapy dropout of HIV/AIDS patients at Mettu Karl Hospital in Illubabor, Ethiopia.

## Main text

### Study area

This study was conducted at Mettu Karl referral Hospital which is found in Ilubabor Zone, Oromia region, southwest part of Ethiopia. This is 600 km far from the capital city of Ethiopia. Mettu is known for its waterfalls such as Sor fall and surrounding evergreen forest.

### Study design

The study was applied a retrospective cohort study design. All patients on antiretroviral therapy from September 2005 up to January 2018 were considered in the study. Secondary data from the Hospital registry was used to retrieve data all about HIV AIDS patients on antiretroviral therapy follow up. There were 3517 patients in a given time interval. Of which a total of 1512 patients were included in the study in a given time interval depending on exclusion criteria (see Additional file 1).

### Variables

The dependent variable is survival time to dropout from the ART starting from September 2005 up to January 2018. The predictor variables were sex, occupation, WHO clinical stage, marital status, baseline regimen type, age, religion, educational level, CD4 level, religion, and body weight.

### Exclusion criteria

Patients with; an incomplete variable of interest, transfer out and death outcomes were excluded from inferential analysis.

### Survival data analysis

Factors associated with predictors of time to dropout from ART were analyzed using Kaplan–Meier comparison and log-logistic regression AFT model. Variables with *p* value < 0.05 was considered statistically significant.

### Kaplan–Meier estimation

The Kaplan–Meier is a nonparametric method used to estimate the survival experience. The survival experience of two or more groups of between-subjects factor can be compared for equality. It is a nonparametric estimator of the survivor function S(t).

$$\hat{S}(t) = \prod\limits_{{t_{J} < t}} {\left(1 - \frac{{d_{j} }}{{n_{j} }}\right)}$$

where $d_{j}$, is the number of individuals who experience the event at time $t_{j}$, and, $n_{j}$ is the number of individuals.

### Log-logistic accelerated failure time model

The log-logistic distribution provides the most commonly used AFT model. The log-logistic regression can exhibit a non-monotonic hazard function which increases at early times and decreases at later times. It is similar in shape to the log-normal distribution but its cumulative distribution function has a simple closed form, which becomes important computationally when fitting data with censoring. The log-logistic survival and hazard function for a log-linear model with no covariates (logT = μ + δε) are;

$${\text{S}}\left( {\text{t}} \right) = \frac{1}{{1 + {\text{e}}^{\theta } {\text{t}}^{\gamma } }}$$

$${\text{H}}\left( {\text{t}} \right) = \frac{{{\text{e}}^{\theta } \gamma {\text{t}}^{\gamma - 1} }}{{1 + {\text{e}}^{{\theta {\text{t}}^{\gamma } }} }}$$

where θ =  $\frac{ - \mu }{\sigma }$ and $\gamma = \frac{1}{\sigma }$ are unknown parameters.

### Results

There were 1512 patients in the cohort study out of which 243 (16.1%) were LTFU. From the total of HIV/AIDS patients, 933 (61.7%) of them were female and 579 (38.3%) were male. The majority of patients 817 (54%) of them were married. From all, 1109 (73.3%) of them were Christians others were Muslim. On the subject of education, 663 (43.8%) of them were primary education complete, 338 (22.4%) of them were secondary education complete, 267 (17.7%) of them were unable to read and write (illiterate), 244 (16.1%) were above secondary education level. Majority of patients 459 (30.4%) were merchants. Of all patients, 520 (34.4%) were started ART at WHO clinical stage three. On the regimen type, there were 120 (7.9%), 488 (32.3%), 493 (32.6%) and 411 (27.2%) patients who took AZT-3TC-EFV, D4t-3TC-NVP, D4t-3TC-EFV and AZT-3TC-NVP medication type respectively. The average age of patients was 33 years and the mean follow up time of patients were 6 years (Table 1).

**Table 1:** Descriptive analysis of variables

N = 1512	Number of events	(%)
Outcome		
Number of dropout	243	16.1
Number of censored	1269	83.9
Sex		
Female	933	61.7
Male	579	38.3
Marital status		
Divorced	188	12.4
Married	817	54.0
Separated	154	10.2
Widow	176	11.6
Never married	177	11.7
Educational level		
Illiterate	267	17.7
Primary school	663	43.8
Secondary school	338	22.4
Above secondary	244	16.1
Religion		
Christian	1109	73.3
Muslim	403	26.7
WHO clinical stage		
Stage I	475	31.4
Stage II	352	23.3
Stage III	520	34.4
Stage IV	165	10.9
Original regimen		
D4t-3TC-NVP	488	32.3
D4t-3TC-EFV	493	32.6
AZT-3TC-NVP	411	27.2
AZT-3TC-EFV	120	7.9
Occupation		
Housewife	344	22.8
Daily labour	296	19.6
Farmer	189	12.5
Government worker	224	14.8
Merchant	459	30.4

From the Chi square test result, dropout was significantly associated with WHO clinical stage (p value = 0.018) and marital status (p-value = 0.007) (see Additional file 2).

### Kaplan–Meier survival estimates

The Kaplan–Meier graph showed that the survival ability of patients marital status married is less than patients with never married (see Additional file 3). From the Kaplan–Meier, log-rank test in Table 2 shows that the survival experience of patients related with occupation and original regimen type status had a significant difference on time to ART dropout at 5% of a significant level.

**Table 2:** Kaplan Meier long rank test result

Variables	Mean estimate			Median estimate			p
	Estimate	95% CI		Estimate	95% CI		
		LCI	UCI		LCI	UCI	
Sex							
Female	182.905	133.502	232.308	135.000	132.501	137.499	0.0889
Male	131.761	119.924	143.597	131.000	124.685	137.315	
Marital							
Divorced	116.161	110.373	121.948	126.000	114.974	137.026	0.0001
Married	148.991	131.994	165.987	135.000	121.870	148.130	
Separated	140.209	134.111	146.308	149.000	128.970	169.030	
Widow	117.070	108.881	125.258	124.000	112.716	135.284	
Never married	171.348	104.636	238.061	130.000	121.385	138.615	
Education							
Illiterate	214.268	165.411	263.126	133.000	124.420	141.580	0.1716
Primary school	137.598	127.585	147.610	135.000	129.390	140.610	
Secondary school	151.528	135.514	167.542	132.000	128.993	135.007	
Above secondary	126.392	117.717	135.068	130.000	117.280	142.720	
Religion							
Christian	160.112	124.299	195.926	132.000	127.944	136.056	0.0694
Muslim	150.494	132.746	168.241	156.000	123.592	188.408	
Occupation							
Housewife	233.275	168.920	297.630	149.000	133.260	164.740	0.0001
Daily labour	137.090	117.988	156.192	130.000	120.881	139.119	
Farmer	122.867	116.163	129.571	138.000	110.609	165.391	
Government worker	117.358	109.166	125.550	118.000	113.796	122.204	
Merchant	125.597	119.114	132.080	129.000	122.795	135.205	
WHO clinical stage							
Stage I	134.390	128.543	140.238				0.8367
Stage II	130.374	123.499	137.250	138.000	115.751	160.249	
Stage III	165.794	125.912	205.675	134.000	131.714	136.286	
Stage IV	144.512	120.156	168.868	133.000	127.310	138.690	
Regimen type							
D4t-3TC-NVP	209.679	156.318	263.041	134.000	129.245	138.755	0.0001
D4t-3TC-EFV	117.646	111.596	123.696	127.000	118.607	135.393	
AZT-3TC-NVP	134.049	129.517	138.581	135.000	129.151	140.849	
AZT-3TC-EFV	125.931	117.382	134.481	123.000	113.594	132.406	

### Model selection

The study used the AIC criterion to compare different models. For each model, the value is computed as AIC = −2 log (likelihood) + 2(p + k). Based on the following statistics value of the AIC/BIC criteria parametric model with log-logistic was preferable for modelling since the smallest value is preferable (see Additional file 4).

From the log-logistic regression model; when a CD4 level added by one unit, the risk of dropout increased by 0.05% (AHR = 1.0005). Likewise, a unit change of weight could accelerate time to dropout by 0.31% (AHR = 1.0031). The risk of dropout of patients with married marital status was 9.8% greater as compared with divorced. Patients ART dropout with separated marital status were at risk as compared to married by 16.82%. The probability of ART dropout with primary education level was 10.58% greater than the illiterate patients. The risks of dropout of patients with daily labour were 87.44% greater than that of housewife. Similarly, the risks to dropout of being farmer were 82.73% as compared to housewife. Being dropout from ART with government worker was increased by 73.72% as compared to a housewife (p < 0.001). Being a merchant also had a negative impact on dropout as compared to housewife. Patients who took D4t-3TC-EFV medication type had a greater risk of dropout as compared to patients who took D4t-3TC-NVP by 84.23% (Table 3).

**Table 3:** Log-logistic AFT model result

Model	AHR	p	95% confidence interval	
Age	1.0034	0.0630	0.9998	1.0070
Marital status				
Divorced (ref)				
Married	1.0980	0.0390	1.0049	1.1999
Separated	1.1682	0.0070	1.0444	1.3067
Widow	0.9323	0.2000	0.8376	1.0377
Never married	1.0987	0.1030	0.9812	1.2302
Education				
Illiterate (ref)				
Primary school	1.1058	0.0110	1.0236	1.1945
Secondary school	1.0526	0.2680	0.9612	1.1527
Above secondary	1.0724	0.2670	0.9480	1.2131
Occupation				
Housewife (ref)				
Daily labour	0.8744	0.0150	0.7848	0.9743
Farmer	0.8273	0.0010	0.7413	0.9233
Government worker	0.7372	0.0001	0.6709	0.8100
Merchant	0.8293	0.0001	0.7656	0.8984
CD4	1.0005	0.0001	1.0003	1.0007
Weight	1.0031	0.0890	0.9995	1.0066
Original regimen				
D4t-3TC-NVP (ref)				
D4t-3TC-EFV	0.8423	0.0001	0.7811	0.9083
AZT-3TC-NVP	1.0467	0.1990	0.9762	1.1222
AZT-3TC-EFV	0.9707	0.5720	0.8757	1.0760

AHR, adjusted hazard ratio; p, p value; Ref, reference category

### Discussion

In this survival retrospective cohort study, there were 243 dropouts from 1512 patients, yielding antiretroviral therapy dropout prevalence were 17/100 patients. In Gambia, only 17.2% dropout was observed [31]. Another study in Nigeria stated that there were 74.9% had been ART dropout which is greater than this investigation [32]. A study which found in sub-Saharan Africa stated that this percentage will vary from 5.7 to 28.9% [33]. A study which was conducted in the region also stated that the percentage of patients dropout was estimated to be up to 31% [34]. The average age of all patients was 33 which is the most productive age group, another study also in Zambia same echo shows that the median age were 34 [35]. Other studies across the country also statement between 31 and 33 [27, 36, 37], which is almost consistent with this study. Even though many manuscript papers stated that age was as a significant factor for antiretroviral therapy dropout, this study explained that age was not a significant impact on antiretroviral therapy dropout. This is inconsistent with findings from other studies [38]. Unlike other studies, weight and WHO clinical stage were not a responsible cause of antiretroviral therapy dropout [39–44]. Patients with higher CD4 level have a greater risk of dropout [AHR = 1.0005 (1.0003–1.0007)], which is directly related with the study in the UK [45] and Hospital of Bergamo cohorts [46], where dropout was related with a higher CD4 count level. Another study in French found that patients with higher CD4 count have increased the risk of antiretroviral therapy dropout [35, 47]. This study stated that sex was not a responsible factor for loss from treatment, but another study in Ethiopia stated that being male was one of the predictors for antiretroviral therapy dropout [48]. Likewise, no association was found between sex and loss from treatment [49–51], but not other studies [52–54]. The difference may arise because of sample size, study design and follow up time difference. Some previous studies suggest that marital status can predict dropout among ART initiators [55–57]. In this data, the patient’s initially receiving D4t-3TC-EFV regimens had decreased risk of dropout as compared with patients who took D4t-3TC-NVP medication type. But the regimen type AZT was not a significant predictor as compared to D4T based which is consistent with another study [57]. This study will serve as resource material for researchers, managers, policymakers. Additionally, the study will be used as a baseline for further researchers.

### Conclusion

In conclusion, HIV/AIDS patients on antiretroviral therapy were dropout in a short period due to patients marital status married and separated, primary education level, high level of CD4 count, being merchants, farmer and daily labour. Investigation on the cause of antiretroviral therapy dropout from a number of HIV/AIDS clinics in the country is highly appreciated.

## Limitations

There were a lot of patients with incomplete records which were excluded from this investigation; this may affect the conclusion of the study.

## Additional files

**Additional file 1: Figure S1.** Sample selection extraction. There were 3517 patients in Mettu Karl Hospital in the time period, But only 1512 patients were included because of variable of interest (patients with lack of adequate information about their follow up were exclude).

**Additional file 2.** Chi-square test result. Test of association between predictor variables and survival status.

**Additional file 3.** Kaplan–Meier survival estimates. The graphical explanation of survival experience ability of patients.

**Additional file 4.** Model comparison. Model comparison means the best fit of data and model to select for conclusion.

## Abbreviations

WHO: World Health Organization
HIV: human immunodeficiency virus
AIDS: acquired immune deficiency syndrome
AFT: accelerated failure time
ART: antiretroviral therapy
