# Supplementary material for: Predictors associated with HIV/AIDS patients dropout from antiretroviral therapy at Mettu Karl Hospital, southwest Ethiopia
Source: BMC Res Notes. 2019 Apr 18;12:232. doi: 10.1186/s13104-019-4267-3 (PMC6471805; doi:10.1186/s13104-019-4267-3)
Supplement: Supplementary file 1 — Additional file 1: Figure S1. Sample selection extraction. There were 3517 patients in Mettu Karl Hospital in the time period, But only 1512 patients were included because of variable of interest (patients with lack of adequate information about their follow up were exclude). [file 13104_2019_4267_MOESM1_ESM.docx]

Total number of patients enrolled in HIV care in Mettu Karl Hospitals from 2005-2018

N= 3,517

Number of patients with death outcome

N=255

The total number of patients with incomplete records

N =1166

The total number of patients with a transfer out outcome status

N=584

The total number of patients in the analysis (N=1512)

Number of dropout/LTFU

N=243

Alive on ART

N=1269

FigureS1. Sample selection extraction
