# Supplementary material for: Predictors associated with HIV/AIDS patients dropout from antiretroviral therapy at Mettu Karl Hospital, southwest Ethiopia
Source: BMC Res Notes. 2019 Apr 18;12:232. doi: 10.1186/s13104-019-4267-3 (PMC6471805; doi:10.1186/s13104-019-4267-3)
Supplement: Supplementary file 2 — Additional file 2. Chi-square test result. Test of association between predictor variables and survival status. [file 13104_2019_4267_MOESM2_ESM.docx]

Chi-square test result

| Variables | Dropout status | | | | | | | | Chi-Square P-value |
| --- | --- | --- | --- | --- | --- | --- | --- | --- | --- |
|  | Dropout | | Censored | | | | | |  |
| Sex |  | |  | | | | | | 0.779 |
| Female | 148 | 60.9% | 785 | | 61.9% | | | |  |
| Male | 95 | 39.1% | 484 | | 38.1% | | | |  |
| Marital Status |  | |  | | | | | | 0.007 |
| Divorced | 39 (16.0%) | | 149 | 11.7% | | | | |  |
| Married | 106(43.6%) | | 711 | 56.0% | | | | |  |
| Separated | 29(11.9%) | | 125 | 9.9% | | | | |  |
| Widow | 38(15.6%) | | 138 | 10.9% | | | | |  |
| Never married | 31(12.8%) | | 146 | 11.5% | | | | |  |
| Educational level |  | |  | | | | | | 0.103 |
| Illiterate | 52 | 21.4% | 215 | | 16.9% | | | |  |
| Primary school | 109 | 44.9% | 554 | | 43.7% | | | |  |
| Secondary school | 54 | 22.2% | 284 | | 22.4% | | | |  |
| Above secondary | 28 | 11.5% | 216 | | 17.0% | | | |  |
| Religion |  | |  | | | | | | 0.122 |
| Christian | 188 | 77.4% | 921 | | 72.6% | | | |  |
| Muslim | 55 | 22.6% | 348 | | 27.4% | | | |  |
| Occupation Status |  |  |  | | | | | | 0.087 |
| Housewife | 50 | 20.6% | 294 | | | 23.2% | | |  |
| Daily labour | 41 | 16.9% | 255 | | | 20.1% | | |  |
| Farmer | 24 | 9.9% | 165 | | | 13.0% | | |  |
| Government worker | 47 | 19.3% | 177 | | | 13.9% | | |  |
| Merchant | 81 | 33.3% | 378 | | | 29.8% | | |  |
| WHO clinical stage |  | |  | | | | | | 0.018 |
| Stage I | 65 | 26.7% | 410 | | | | 32.3% | |  |
| Stage II | 51 | 21.0% | 301 | | | | 23.7% | |  |
| Stage III | 105 | 43.2% | 415 | | | | 32.7% | |  |
| Stage IV | 22 | 9.1% | 143 | | | | 11.3% | |  |
| Original regimen |  |  |  | | | | | | 0.099 |
| D4t-3TC-NVP | 88 | 36.2% | 400 | | | | | 31.5% |  |
| D4t-3TC-EFV | 64 | 26.3% | 429 | | | | | 33.8% |  |
| AZT-3TC-NVP | 67 | 27.6% | 344 | | | | | 27.1% |  |
| AZT-3TC-EFV | 24 | 9.9% | 96 | | | | | 7.6% |  |
