# Supplementary material for: Predictors associated with HIV/AIDS patients dropout from antiretroviral therapy at Mettu Karl Hospital, southwest Ethiopia
Source: BMC Res Notes. 2019 Apr 18;12:232. doi: 10.1186/s13104-019-4267-3 (PMC6471805; doi:10.1186/s13104-019-4267-3)
Supplement: Supplementary file 4 — Additional file 4. Model comparison. Model comparison means the best fit of data and model to select for conclusion. [file 13104_2019_4267_MOESM4_ESM.docx]

Model comparison

|  | Parametric model | | Gamma Frailty model | | Cox model | |
| --- | --- | --- | --- | --- | --- | --- |
| Type of distribution | AIC | BIC | AIC | BIC | AIC | BIC |
| log-logistic | 662.2073 | 763.3099 | 663.6981 | 770.1219 | 2737.149 | 2827.61 |
| Lognormal | 678.2422 | 779.3448 | 680.2236 | 786.6474 |  |  |
| exponential | 1226.737 | 1322.518 | 1228.737 | 1329.839 |  |  |
| Weibull | 705.1167 | 806.2193 | 664.2034 | 770.6272 |  |  |
| gamma | 676.2009 | 782.6247 |  |  |  |  |

*Note: AIC= Akaike information criterion, BIC=Bayesian information criterion*
