# Supplementary material for: Predictors associated with HIV/AIDS patients dropout from antiretroviral therapy at Mettu Karl Hospital, southwest Ethiopia
Source: BMC Res Notes. 2019 Apr 18;12:232. doi: 10.1186/s13104-019-4267-3 (PMC6471805; doi:10.1186/s13104-019-4267-3)
Supplement: Supplementary file 4 [file 13104_2019_4267_MOESM3_ESM.docx]

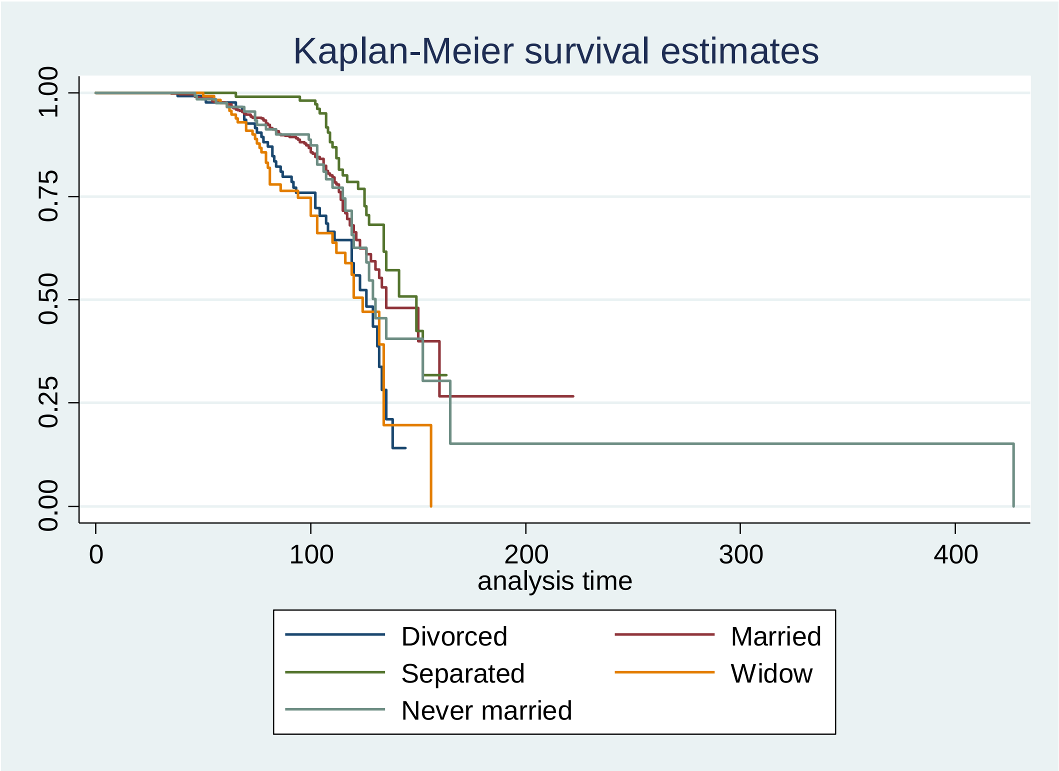
Kaplan-Meier survival estimates
1.00
0.75
0.50
0.25
0.00
0
100
200
300
400
analysis time
Divorced
Married
Separated
Widow
Never married
